# Central sleep apnea: misunderstood and mistreated!

**DOI:** 10.12688/f1000research.18358.1

**Published:** 2019-06-28

**Authors:** Jerome A. Dempsey

**Affiliations:** 1 Department of Population Health Sciences, University of Wisconsin - Madison, WARF Building, 7th Floor, 614 Walnut Street, Madison, WI 53726, USA

**Keywords:** Heart failure, hypoxic exposure, opioid use, loop gain, positive airway pressure

## Abstract

Central sleep apnea is prevalent in patients with heart failure, healthy individuals at high altitudes, and chronic opiate users and in the initiation of “mixed” (that is, central plus obstructive apneas). This brief review focuses on (a) the causes of repetitive, cyclical central apneas as mediated primarily through enhanced sensitivities in the respiratory control system and (b) treatment of central sleep apnea through modification of key components of neurochemical control as opposed to the current universal use of positive airway pressure.

## Introduction

**Figure 1.**
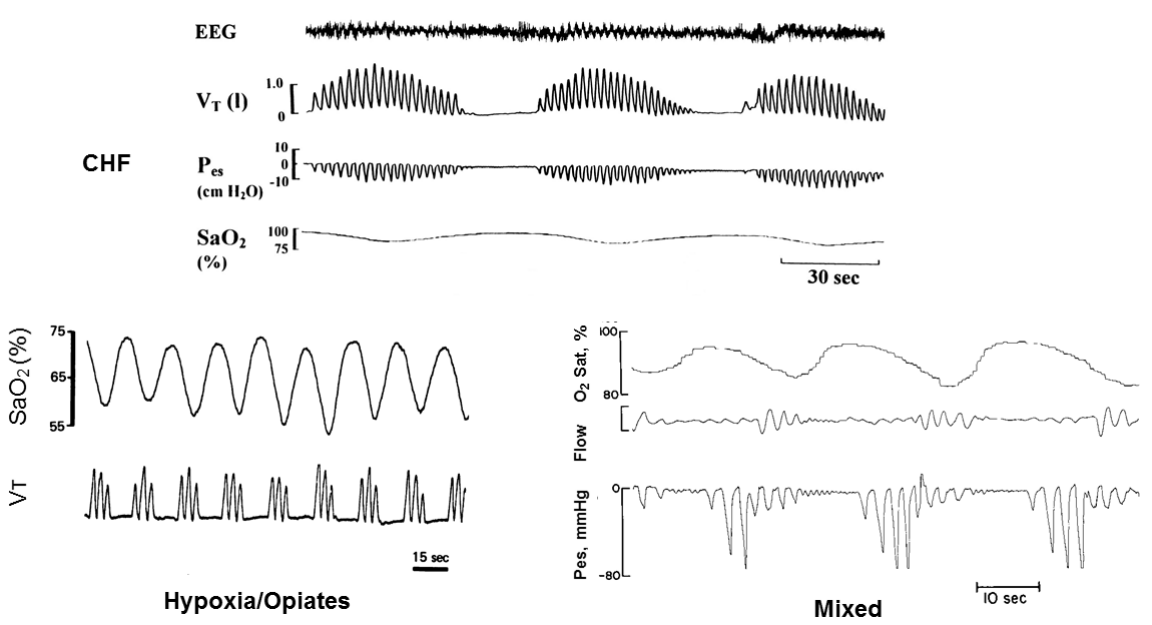
Three common types of cyclical central sleep apneas. The three types are congestive heart failure (CHF), high-altitude/chronic opioid use, and mixed-central followed by obstructive apneas. See text for detailed descriptions. EEG, electroencephalography; P _es_, esophageal pressure; SaO _2_, arterial oxygenated hemoglobin saturation; Vt, tidal volume. Adapted from ^15^.

Central sleep apneas (CSAs) occur when there is a transient reduction by the ponto-medullary respiratory rhythm generator. In contrast, obstructive sleep apnea (OSA) involves continuous respiratory efforts made against a closed airway. The four major types of sleep apnea are depicted in Figure 1. Heart failure patients with left ventricular dysfunction—with or without preserved ejection fraction—are the most prevalent type of CSA and over one half of these patients show sleep-disordered breathing (apnea/hypopnea index [AHI] greater than 15 events per hour of sleep) ^1, 2^. The periodic waxing and waning of tidal volume (Vt) followed by apneas of at least 10 seconds’ duration accompanied by intermittent hypoxemia and transient cortical electroencephalography arousal are typical of non-rapid eye movement (NREM) sleep in congestive heart failure (CHF) with periodic cycles of at least 50 seconds. CSA and “cluster type” periodic breathing with short periodic cycles (10–25 seconds) are common in healthy sojourners during NREM sleep at high altitudes (>3000 m, oxygen saturation [SaO _2_] of about 90%) and even at more moderate high altitudes in susceptible people, especially with prolonged residencies ^3, 4^. This cluster-type periodic breathing with relatively short cycles is also common at sea level in over half of chronic opioid users, and the severity of CSA is proportional to opiate dose ^5^. A third type of CSAs consists of those immediately preceding an airway obstruction. In the example given here ( Figure 1, mixed), esophageal pressure is used as a highly sensitive means of distinguishing central (no inspiratory effort) from obstructive (increasing inspiratory effort) apneas. In clinical practice, less sensitive, indirect measures of inspiratory effort are used, thereby likely underestimating the prevalence of mixed apneas. Even more difficult detection problems occur in attempts to distinguish hypopneas of central versus obstructive origin. The measurements of suprasternal pressure or “shape” (or both) of the nasal pressure waveform during inspiration have shown favorable comparisons with esophageal pressure as a marker for increased upper airway resistance ^6, 7^ and should be explored further for use in the clinical polysomnogram.

## Central sleep apnea sequelae

Cyclical CSAs—like their obstructive counterparts—are pro-inflammatory with substantial long-term deleterious effects during wakefulness, including enhanced sympathetic vasomotor outflow and vascular endothelial dysfunction, neurocognitive deficits, and insulin insensitivity ^2^. Both the repeated arousals and especially the chronic intermittent hypoxemia (CIH) attending cyclical CSA have been implicated. However, when supplemental O _2_ was used to eliminate the intermittent hypoxemia accompanying airway obstruction in a sleeping canine model, the transient arousals by themselves were not sufficient to elicit the significant increase in daytime mean arterial pressure observed when both CIH and arousals accompanied the OSA ^8^.

The amount of oxygenated hemoglobin (HbO _2_) desaturation for any given apnea length will depend upon the starting (pre-apneic) position on the sigmoid HbO _2_ dissociation curve, the end-expiratory lung volume at which the apnea begins, and the individual’s oxygen consumption, cardiac output, and resultant arterial-to-venous O _2_ content difference ^9, 10^. Importantly, the fast reoxygenation phase at apnea termination—as occurs in patients with sleep apnea—was found to be especially pro-inflammatory ^11^. The molecular basis for the highly inflammatory response to intermittent (as opposed to constant) hypoxemia has been explained by Semenza and Prabhakar as an upregulation of both pro- and anti-oxidant transcription factors, hypoxia-inducible factor (HIF) 1α and 2α in constant hypoxemia but a suppression of the anti-oxidant HIF 2α in intermittent hypoxemia ^13^. On the other hand, intermittent hypoxemia induced experimentally at very mild levels and for cycles that are several minutes in duration and for very brief periods of daily administration elicits a significant plasticity in phrenic motor neurons ^14^. This adaptive response to induced mild, brief CIH is in sharp contrast to that elicited via repetitive sleep apneas; the latter are unlikely to convey any significant beneficial biological benefit.

The amount of sleep apnea that may be “clinically significant” remains controversial; correlational studies claim that a frequency of as few as 5 to 10 events per hour has significant chronic cardiovascular consequences ^15^. However, these claims have not been tested via interventional treatments in people with these lower levels of AHI. Alternatively, given the relative importance of the intermittent hypoxemia insult, it seems appropriate to define the severity of sleep-disordered breathing by using indices that are based on the degree of intermittent hypoxemia incurred ^2, 12^.

## Mechanisms common to all types of central sleep apneas

### Removal of the wakefulness drive to breathe

The transient cessation of the medullary respiratory pattern generator neurons requires an unmasking of a sensitized apneic threshold in NREM sleep, as induced by a transient ventilatory overshoot involving both mild to moderate hypocapnia plus augmented Vt values. Carotid body denervation studies in rodents and canines demonstrate that the carotid bodies are required for sensing the low partial pressure of carbon dioxide (PaCO _2_) and causing ventilatory instability and cyclical apneas ^16, 17^. However, studies in the sleeping canine with isolated, perfused carotid chemoreceptors also showed that hypocapnia induced only at the level of either peripheral or central chemoreceptors was insufficient to elicit apnea ^18, 19^ and that hypocapnia induced at the level of the isolated, perfused carotid chemoreceptor caused a marked inhibitory effect on central CO _2_ sensitivity. Thus, interdependence of function between peripheral and central chemoreceptors was an essential mediator of the apnea elicited via transient hypocapnia ^18– 20^. Furthermore, vagal blockade in sleeping animals showed that inhibitory feedback from the lung stretch accompanying transient increases in Vt also contributes to the apnea following a ventilatory overshoot ^21^.

These inhibitory effects on breathing are opposed by excitatory central short-term potentiation mechanisms which preserve ventilatory drive immediately following chemoreceptor-driven ventilatory overshoots while awake but apparently not sufficiently to prevent apnea or hypopnea during NREM sleep ^22, 23^. So clearly, a significant “wakefulness drive” to breathe exists. It is manifested in sleep-induced inhibition of medullary inspiratory activity as well as withdrawal of tonic hypoglossal neuronal activity ^24, 25^ and removing it in NREM sleep also appears to compromise the control system’s vigilance in protecting against chemo- and mechanoreflex-induced apneas. An additional example of an NREM sleep-induced compromise of control system vigilance occurs with respiratory compensation for loads induced by increased airway resistance which occur immediately to preserve ventilation during wakefulness but are absent in NREM sleep ^26^. Groups of neurons within the ponto-medullary axis which might mediate these influences of wakefulness on respiratory control have been postulated, although definitive evidence on this complex problem remains elusive ^25^.

Two additional mechanisms to enhance post-apneic ventilatory overshoots include the following: (a) apneas are commonly prolonged until PaCO _2_ rises above its normal pre-apneic, eupneic level ^27^; and (b) transient arousals at end apnea are common and will enhance the magnitude of the transient ventilatory overshoot response to chemoreceptor stimulation.

### High loop gain

The unmasking of these reflex mechanisms underlies a sleep-induced central apnea. However, the repeated cyclical occurrence of transient ventilatory undershoots (apneas/hypopneas) and overshoots requires that respiratory control system “loop gain” to be elevated. Loop gain is a dynamic measure of how close a physiologic control system is to instability.

$$\begin{array}{l} \text{Loop}\;\text{Gain}\;=\;\text{controller}\;\text{gain}\;\times \;\text{plant}\;\text{gain}\\ \text{Loop}\;\text{Gain}\;=\left[\begin{array}{c} \text{ΔVE}/\text{Δ}PaCO2\\ \text{(>}\;\text{<}\;eupnea\text{)} \end{array}\right]\;\times \;\left[\frac{\text{PaCO2}}{\text{lung}\;\text{vol}\;}\cdot \begin{array}{c} \text{transit}\;\text{time}\\ \text{delay} \end{array}\right] \end{array}$$

**Figure 2.**
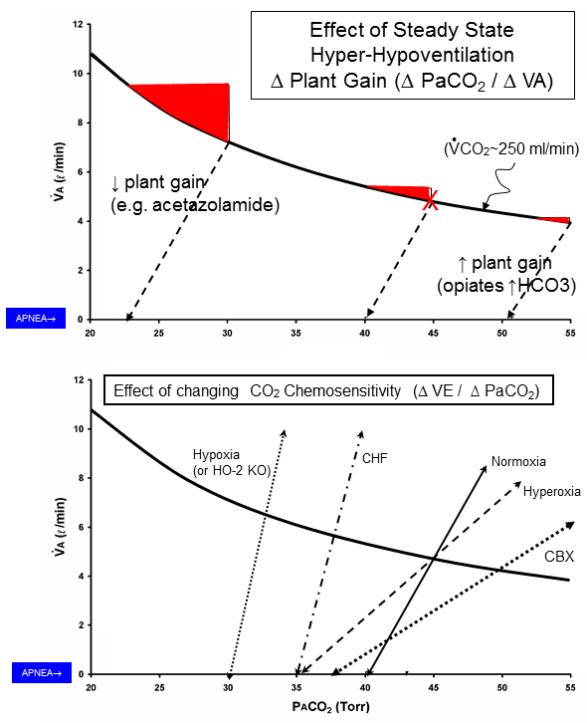
Diagram of alveolar gas equation to illustrate the effects of loop gain components on the propensity for central and cyclical central sleep apnea. The equation is PaCO _2_ = V̇CO _2_/V̇A·K, where V̇CO _2_ = 250 mL/min. Each example shown is from an experimental study in sleeping humans or canines in which the apneic threshold and the slope of the carbon dioxide (CO _2_) response below eupnea were measured during non-rapid eye movement (NREM) sleep by using a mechanical ventilator in the assist-control mode to gradually raise tidal volume (Vt) and lower partial pressure of end-tidal carbon dioxide (PetCO _2_) until apnea occurred. The top panel shows effects of changing “plant” gain (ΔPaCO _2_/ΔV̇A) with steady-state hyper- or hypo-ventilation along the iso-metabolic hyperbola. The red filled-in areas indicate the magnitude of increase in alveolar ventilation needed to reduce PaCO _2_ sufficiently to reach the apneic threshold. For example, under control conditions in NREM sleep (eupneic PaCO _2_ ~ 45 mm Hg, denoted by X), a transient ventilatory overshoot of about 1 L/min is required to reduce PaCO _2_ ~ 5 mm Hg to the apneic threshold of 40 mm Hg. With steady-state hyperventilation (for example, oral acetazolamide; PaCO _2_ 30 mm Hg), the required ventilatory overshoot to achieve apnea (PaCO _2_ 23 mm Hg) is about twice that of the control; conversely, with steady-state hypoventilation (for example, metabolic alkalosis, opiate use; PaCO _2_ ~ 55 mm Hg), the required ventilatory overshoot to achieve apnea (PaCO _2_ ~ 51 mm Hg) is about one third that of control. Not illustrated here are ( **a**) the effects of transient arousal from sleep, which will increase the magnitude of the ventilatory overshoot above eupnea, and ( **b**) the dynamic effects of lung volume on plant gain. For example, at low lung volumes, plant gain is raised; thus, the CO _2_ washout from the alveoli will occur more quickly and will require smaller transient increments in ventilation to reach the apneic threshold ^31^. The lower panel shows effects of changing “controller” gain or chemoreceptor sensitivity to PCO _2_ (ΔV̇A/ΔPaCO _2_) above eupnea (which affects the magnitude of the ventilatory overshoot) and below eupnea (which affects the CO _2_ “reserve” or difference in PaCO _2_ between eupneic breathing and the apneic threshold). Note the increased chemosensitivities of the CO _2_ response slopes above and below eupnea in congestive heart failure (CHF) and hypoxic environments and the reduced CO _2_ sensitivity in hyperoxia which is further reduced with carotid chemoreceptor denervation (see text). CBX, Carotid Body Denervation; K, constant .863; PaCO _2_, mmHg arterial PCO2; PCO _2_, partial pressure CO2; V̇A, alveolar ventilation ; V̇CO _2_ ventilation to CO2 production; VE, ventilation.

The principal component of a high loop gain is an excessive chemosensitivity to CO _2_ both above and below the level of eupneic ventilation. This high gain means that both ventilatory undershoots in response to a transient hypocapnia and ventilatory overshoots in response to a combination of apnea-induced hypoxemic and hypercapnic chemoreceptor stimuli are excessive, thereby precipitating the continued breathing periodicity ^28, 29^. The concept of loop gain and its two principal components—controller (CO _2_ chemosensitivity or ΔVE/ΔPaCO _2_ slope) and plant (or ΔPaCO _2_/ΔVE) gain—are illustrated in Figure 2. (We acknowledge that the concept of loop gain was developed to characterize the dynamic behavior of linear “systems…yet substantial non-linearities exist in virtually all components of the respiratory control system, especially when apnea occurs in the hypocapnic range” ^29^. Furthermore, our schematic of loop gain in Figure 2 portrays only steady-state conditions for variables in the alveolar gas equation. Despite these limitations, considerable experimental evidence has accumulated in sleeping animals and humans to demonstrate that altering one or more components of loop gain elicits predictable influences on ventilatory stability/instability. See text and Figure 2.)

Finally, another common feature of all types of central apneas is their predominance in NREM sleep and especially in lighter sleep stages as well as their relative scarcity in phasic REM sleep ^1^. Coincidentally, the apneic threshold which resides within 2 to 5 mm Hg PaCO _2_ below normal awake levels in healthy individuals in NREM is not readily demonstrable in phasic REM sleep ^30^. The ventilatory response above eupnea to added CO _2_ is also blunted in NREM (versus wakefulness) in part due to a truly reduced CO _2_ chemoreceptor sensitivity ^32^ but also to loss of tonic neural motor input to pharyngeal dilator muscles resulting in increased upper airway resistance ^33, 34^. Transient arousal at apnea termination temporarily restores this tonic input and reduces airway resistance, thereby contributing to ventilatory overshoot prior to sleep restoration. Similarly, in REM sleep, ventilatory responsiveness to CO _2_ above eupnea is reduced in slope and shows an almost random Vt or diaphragmatic electromyography responses (or both) to increasing PaCO _2_ (with or without coincident airway occlusion) rather than an orderly dose response as in quiet wakefulness or NREM sleep ^35^. Perhaps the erratic, sporadic increases in medullary inspiratory neuronal drive as observed during phasic REM in the sleeping cat override hypocapnic inhibition or hypercapnic stimulation of central respiratory motor output ^36^.

## Central sleep apnea in congestive heart failure

The key characteristics of CHF present an almost “perfect storm” to promote periodic breathing. We summarize the following key contributions:

- Controller (and therefore loop) gain is elevated because of enhanced carotid chemosensitivity, leading to a highly sensitized apneic threshold (within 1 to 2 mm Hg of eupneic PaCO _2_) and an enhanced ventilatory overshoot to transient reductions in ventilation ^37^. In turn, the pioneering work of Schultz *et al*. in animal models of CHF has demonstrated the critical importance of a reduced carotid sinus blood flow and shear stress in eliciting the enhanced carotid chemosensitivity ^38, 39^.
- Elevated pulmonary vascular pressures in CHF—especially during recumbency with central fluid shifts ^40^ —contribute to lung C fiber and ventilatory stimulation, an increased CO _2_ response slope sensitivity, and a narrowed CO _2_ reserve ^41^. This “extra respiratory stimulus” might also prevent mild hypoventilation and CO _2_ retention in CHF which normally occurs at sleep onset ^37^.
- Patients with CHF commonly have a compromised vasodilatory and vasoconstrictive cerebrovascular response to transient hypercapnia and hypocapnia, respectively ^42^. This means that medullary CO _2_ and H ^+^ levels are less protected and more labile in response to transient changes in systemic PaCO _2_; thus, the ventilatory response slopes to ΔPaCO _2_ above and below eupnea are sensitized.
- Low cardiac output in CHF means prolonged circulation times, thereby prolonging the delivery of altered blood gases from lung to carotid body (CB) and prolonging the periodic respiratory cycles.

A common misconception particularly with reference to causes of periodic breathing in CHF is that a reduced steady-state PaCO _2_ will precipitate periodic breathing, presumably because the patient’s eupneic PaCO _2_ is moved closer to their apneic threshold ^43, 44^. This is a misconception because (as shown in Figure 2) the position of the apneic threshold relative to eupneic PaCO _2_ is labile; that is, the CO _2_ reserve below eupnea is determined by the chemosensitivity and slope of the CO _2_ response ^45^. Importantly, a reduced steady-state PaCO _2_ actually reduces plant gain, meaning that a larger transient ventilatory response is required to lower PaCO _2_ to reach the apneic threshold ( Figure 2, top panel). Thus, the reduced steady-state PaCO _2_ is an important protector against apnea; alternatively, steady-state hypoventilation and hypercapnia elevate plant gain and precipitate ventilatory instability ^45^ (also see examples under the “Non-positive airway pressure (PAP) treatments of CSA…” section below).

## Central sleep apnea in hypoxic environments

Most sojourners with a wide spectrum of hypoxic chemosensitivity experience periodic breathing in hypoxic environments ^4^; exceptions include healthy high-altitude natives with markedly depressed hypoxic chemosensitivity ^46^. In the sojourner, the development of periodic breathing during NREM sleep in hypoxia occurs within a few minutes of hypoxic onset as an initial hyperventilation and reduced PaCO _2_ evolve into progressively larger hyperpnea/hypopnea oscillations in Vt and then—following an augmented inspiration when PaCO _2_ reaches the apneic threshold—expiratory time is prolonged and hyperpnea/apnea combinations ensue with a cycle period of 20 to 25 seconds. Loop gain is increased in hypoxia because chemoreceptor CO _2_ sensitivity is increased more than plant gain is reduced ( Figure 2). Thus, the apneic threshold resides within 1 to 2 mm Hg of the eupneic PaCO _2_ during NREM sleep, ensuring a significant ventilatory undershoot with even small levels of transient hypocapnia ^47^. Furthermore, the ventilatory overshoot at apnea terminations is amplified because of the synergistic stimulatory effects on carotid chemoreceptors of hypoxemia plus hypercapnia (that is, asphyxia). Chemoreceptor sensory inputs drive both medullary rhythm-generating neurons and arousal-producing cortical neurons ^4, 29^. These combined oscillating powerful drives and inhibitors to breathing likely explain, respectively, the abrupt large ventilatory overshoots at apnea termination and abrupt ventilatory undershoots at end hyperpnea in hypoxic environments, resulting in the cluster-type breathing pattern ( Figure 1).

## Opiate-induced central sleep apnea

Respiratory depression, first with hypoventilation and then outright apneas, occurs because of the actions of opioids on mu and kappa opioid receptors, and the most profound respiratory depression effects occur during a background of anesthesia and NREM sleep ^5^. Opioid receptors located on both the rhythm-generating medullary neurons in the pre-Bötzinger and RTN/pFRG complexes are inhibited, and both hypoxic and hypercapnic ventilatory response sensitivities are depressed upon acute intravenous or long-term oral opioid administration. Hypoglossal motor neurons are also depressed with high doses of opioids and upper airway resistance is increased ^5, 48– 51^.

The dilemma in understanding the role of an enhanced loop gain in opioid-induced periodic breathing is that—unlike those cases with CHF or in hypoxia—periodic breathing occurs in the face of depressed central respiratory neurons and compromised chemosensitivity. So, although we might expect apnea to occur at sleep onset with opioid use, there is no mechanism for repeated cyclical apneas based on the model of increased controller and loop gains. One possibility here is an increased plant gain secondary to opioid-induced steady-state hypoventilation and CO _2_ retention during sleep with subsequent displacement of ventilation and PaCO _2_ down the isometabolic hyperbola ( Figure 2, top panel). This means that the apneic threshold will be reached with extremely small transient increases in alveolar ventilation ^45^. The probability of steady-state hypoventilation has been documented indirectly in some chronic opioid users during wakefulness and sleep ^52^, but more studies quantifying CO _2_ retention need to be conducted during sleep in order to determine whether there is a significant potential role for increased plant gain. If the higher plant gain was responsible for initiating apneas and some periodicity in chronic opioid users, CIH over time might be expected to sensitize the peripheral chemoreceptors and thereby exacerbate the periodic breathing in a positive feed-forward fashion ^50^.

## Mixed: central/obstructive apneas

Centrally induced periodicities and apneas occurring in patients with underlying collapsible airways will elicit repeated obstructions at the nadir of an oscillating central respiratory motor output during sleep ^53– 55^. The neurophysiologic basis for this effect of central respiratory motor output on central and obstructive apnea resides in the influence of efferent central respiratory motor output on activation/inhibition of motor neurons serving both upper airway pharyngeal dilator musculature and the chest wall respiratory pump musculature ^33^. Accordingly, it is common to see central, obstructive, and mixed apneas in patients with CHF, even within the same night, possibly secondary to factors such as fluid accumulation around the upper airway and changes in head, neck, and body position ^2, 56^. Also, predominantly patients with OSA acquire CSA as chemoreceptor sensitivity increases and central respiratory motor output instability occurs during sojourn at high altitudes ^57^. Similarly, a residual CSA is often unmasked in patients with OSA during the early stages of continuous PAP (CPAP) treatment (that is, so-called “complex” sleep apnea). An increased prevalence of OSA (as well as CSA) in chronic opioid use ^58^ may also reflect central instabilities in respiratory motor output superimposed on a collapsible airway.

## Central sleep apnea treatment via positive airway pressure

The use of PAP is routinely the clinician’s first—and often only—treatment choice for any type of sleep-disordered breathing. However, evidence to date shows that this is not always the best approach for cases of predominant CSA. CPAP treats less than half of central apneas effectively and when it is applied in large clinical trials in patients with CHF, significant reductions in CSA did not appear in most patients until after the first several months of treatment. Patient survival was eventually compromised in these trials ^2, 56, 59^. Low adherence to CPAP is always a major problem, especially if the failure to use CPAP occurs in the early morning hours when REM sleep is prevalent and accompanied by high levels of sympathetic nerve activity and cardiovascular stress ^60, 61^. Theoretically, assisted servo-ventilation should be an ideal approach to treat CSA, mixed apnea, and OSA because it delivers inspiratory pressure support to increase flow rate in the face of hypoventilation, also, it titrates expiratory PAP to eliminate airway obstructions ^2, 56^. However, to date, adaptive servo-ventilation (ASV) produced no survival benefit and actually increased mortality in CHF patients with CSA ^2, 56^. Speculation on the reasons for this ASV failure points to potential deleterious effects of PAP on left ventricular function, especially if the ASV device imposes excessive intrathoracic pressures in vulnerable patients ^2^. More thorough study of the effects of varying levels and types of PAP on left and right heart function is clearly indicated! In addition, alternative treatments to PAP must be explored.

## Non-positive airway pressure treatments of central sleep apnea aimed at ameliorating one or more components of high loop gain

### Congestive heart failure

The use of nocturnal supplemental O _2_ in the mildly hyperoxic range addresses the primary problem of excessive chemoreceptor sensitivity and has successfully reduced AHI and eliminated CIH in several descriptive studies using relatively small numbers of patients with CHF ^2, 62, 63^. The durations of remaining apneas are lengthened. In select cases of OSA plus CSA, supplemental O _2_ is also effective by itself ^64– 66^ or in combination with CPAP or ASV devices ^66, 67^.

Oral acetazolamide will reduce plant gain to varying extents, depending upon the magnitude of the coincident reduction in PaCO _2_, and eliminate significant amounts of CSA and also reduce fluid load and associated airway compression via its diuretic actions ^2, 68^. Rebreathing added dead space with less than a 2 mm Hg increase in partial pressure of end-tidal carbon dioxide (PetCO _2_) reduces plant gain and markedly lowers CSA, transient arousals, and CIH in patients with ****CHF ^44, 69^; even OSA is markedly reduced via supplemental CO _2_ in select cases ^64, 70^. Importantly, augmenting central respiratory motor drive to reduce CSA must also weigh the potential negative effects of raising chemosensory input on arousals, sleep state, or autonomic cardiovascular regulation (or a combination of these) which may occur if the imposed hypercapnia is excessive ^71^.

Exercise training has also been shown to reduce chemosensitivity in animal models and patients with CHF ^72, 73^, presumably because of the intermittent increase in blood flow and shear stress in the carotid bifurcation ^39^. Chronic statin therapy in rodents with CHF has also shown promise as a means of reducing chemosensitivity and CSA ^74^.

It is clearly time to introduce clinical trials using these non-PAP approaches—alone or in combination—to reducing loop gain ^2^. Hopefully, such trials will also include assessments of loop gain components, arousal threshold sensitivity, and airway collapsibility so that investigators will have the tools to determine the causes of inter-individual variations in response to these proposed treatments ^64, 67^. To address future potential treatments, we note that pharmacologic and even gene transfer approaches are under development in animal models for reducing carotid chemoreceptor hypersensitivity. Specific targets include downregulation of carotid chemoreceptor excitatory neurotransmitters or adrenergic receptors as well as upregulation of shear stress–sensitive transcription factors to enhance carotid sinus blood flow ^39, 75, 76^.

### Hypoxic environments

PAP administered via servo-ventilator or CPAP was not consistently effective in reducing CSA in hypoxia ^57, 77^. Nocturnal administration of supplemental O _2_ sufficient to return SaO _2_ within 2 to 3% of sea level values eliminated periodic breathing at high altitudes almost immediately ^4, 77^. Oral acetazolamide elicits steady-state hyperventilation and reduces PaCO _2_ and loop gain, thereby reducing periodic breathing, in sleeping sojourners, whereas adding 1 to 2 mm Hg PaCO _2_—via increased fractional inspired carbon dioxide (FiCO _2_)—is sufficient to completely eliminate periodic breathing via reduced plant gain ^3, 4^. OSA patients sojourning at even moderate altitudes experience periodic CSA and adding oral acetazolamide to their CPAP treatment significantly reduced both central and obstructive apneas ^57^. A limited amount of data have shown that these various means of reducing sleep-disordered breathing at high altitude also substantially reduce periodic transient arousals from sleep, increase the time spent in deep sleep, and diminish the carryover daytime sequelae of periodic breathing and CIH, such as increased systemic blood pressure, fatigue, neurocognitive impairment, and hypersomnolence ^78, 79^. The effect of preventing periodic breathing in hypoxia on nocturnal and daytime systemic and pulmonary vascular resistance at rest and during exercise needs to be addressed.

### Chronic opiate use-induced sleep-disordered breathing

The aim here is to treat sleep-induced hypoventilation and any associated increase in airway resistance and apneic and hypopneic events. Opioid withdrawal or dose reductions in chronic users eliminates or significantly lowers the sleep-disordered breathing and CIH ^80^. Again, CPAP is not an effective means of treatment; however, non-invasive positive pressure ventilation devices with bilateral pressure support and backup respiratory rates should be ideal for this treatment. Accordingly, adaptive servo-ventilators were substantially more effective than CPAP in reducing sleep-disordered breathing and arousals in chronic opioid users with a relatively high adherence rate (compared with CPAP) followed over several months ^48^. Increasing the (suspected) reduced ventilatory drive and hypoventilation during sleep with opiate users might be achieved by reducing plant gain via ventilatory stimulation using oral acetazolamide or via dead space rebreathing just sufficiently to raise PetCO _2_ 1 to 2 mm Hg. To date, these approaches to raise central respiratory motor output during sleep have not been attempted in chronic opioid users with CSA.

## Summary

CSA either by itself or in combination with obstructive apnea is not uncommon among patients with sleep-disordered breathing, especially in heart failure, at high altitudes, and with chronic opioid use. The CIH accompanying CSA elicits cardiovascular consequences which carry over to the waking state. The underlying causes of CSA are complex and not completely understood, although increased loop gain is now well established as a common feature. Treating CSA effectively and safely has had little success to date, presumably because CPAP has been the automatic treatment of choice among sleep medicine clinicians. Accordingly, it is time to match treatments with causative mechanisms but with the understanding that not all patients with CSA will share the same underlying causes.
